# The Exciting New Field of HER2-Low Breast Cancer Treatment

**DOI:** 10.3390/cancers13051015

**Published:** 2021-03-01

**Authors:** Daniel Eiger, Elisa Agostinetto, Rita Saúde-Conde, Evandro de Azambuja

**Affiliations:** 1 Academic Promoting Team, Institut Jules Bordet, L’Universite Libre de Bruxelles (U.L.B.), 1000 Brussels, Belgium; danieleiger@gmail.com (D.E.); elisa.agostinetto@bordet.be (E.A.); rconde@ipolisboa.min-saude.pt (R.S.-C.); 2 Medical Oncology and Haematology Unit, Humanitas Cancer Center, Humanitas Clinical and Research Center—IRCCS, Rozzano, 20089 Milan, Italy; 3 Medical Oncology Department, Instituto Português de Oncologia de Lisboa Francisco Gentil, 1099-023 Lisbon, Portugal

**Keywords:** HER2-low breast cancer, trastuzumab, antibody–drug conjugates, trastuzumab–deruxtecan, trastuzumab–duocarmazine, zenocutuzumab

## Abstract

**Simple Summary:**

Breast cancer can express, at varied levels, a protein named HER2, commonly responsible for making it grow and send distant metastases. In the past, patients affected by the so called HER2-positive breast cancer had lower probabilities of cure and survival, though with the advent of drugs that target HER2, three decades ago, their prognosis has greatly improved. So far, only patients with strong HER2 expression on their tumour can be treated with these benefitial drugs, like trastuzumab, though recently stronger drugs have also been shown capable of eliminating breast cancer cells with lower levels of HER2 expression (HER2-low). Sooner or later, these new drugs, like trastuzumab-deruxtecan, may be available for treating such patients. Therefore, the aim of this narrative review of the literature is to provide an outline of what is going on on this specific field of research, and what could be expected in the future in the clinic.

**Abstract:**

Since human epidermal growth factor receptor-2 (HER2) characterization, going through clinical research and regulatory approval of HER2-targeted therapies, much has elapsed and is still unfolding. Hitherto, only breast cancer (BC) patients with HER2 immunohistochemistry 3+ or with HER2 gene fluorescence in-situ hybridization (FISH) amplification (a.k.a., HER2-positive BC) have benefited from anti-HER2 agents. In recent years, however, much of the research effort has been expanded, with positive outcomes being reached for formerly known HER2-negative BC that yet express HER2 to some degree (HER2 immunohistochemistry 1+ or 2+, but FISH negative) and are currently being classified as HER2-low BC for the purpose of trial enrollment. In this sense, our aim is to review the body of evidence of HER2-low BC that led to the study of first-generation anti-HER2 agents, like trastuzumab, and how they have failed to achieve any clinical applicability in this setting. In addition, we review new data that is leading to the growing success of the new generation of drugs, especially the promising HER2-directed antibody–drug conjugates. A narrative review is also performed regarding the rationale behind the consolidated and ongoing clinical trials studying anti-HER2 agents in combination with unrelated agents, such as immunotherapy, endocrine therapy, and CDK4/6 inhibitors. Hopefully, all this ongoing research effort will be able to extend the survival benefits seen with anti-HER2 agents in HER2-positive disease, at least to some degree, to the greater proportion of patients with HER2-low BC.

## 1. Introduction

Drugs targeting the human epidermal growth factor receptor-2 (HER2) have revolutionized the treatment landscape of HER2-positive breast cancer (BC) patients, creating a new standard of remarkable survival outcomes for once a BC subtype with gloomy perspectives [1,2,3,4,5]. Early trials testing trastuzumab, the first anti-HER2 drug ever approved, demonstrated that tumor responses were restricted to patients whose tumors stained 3+ for HER2 on immunohistochemistry (IHC) or stained 2+ but had HER2 gene amplification (≥2 copies) on fluorescence in-situ hybridization (FISH) [6,7,8]. These early observations have established the standard to which subsequent trials and international guidelines would test and recommend anti-HER2 therapies, respectively [9,10,11,12]. While HER2-positive BC comprises only around 20% of newly diagnosed cases, a greater proportion of patients (≈40–50%) have BC categorized as HER2-low, i.e., IHC of 1+ or 2+ but FISH negative [13,14,15]. Nonetheless, HER2-low BC is considered altogether with those with 0+ at IHC as HER2-negative, for the purpose of current treatment decisions (i.e., non-eligible for anti-HER2 therapies) [14,15,16].

Patients with HER2-low BC spam a heterogeneous group, immunohistochemically comprised of a majority of hormone receptor (HR) positive tumors (65–83%), while the rest has HR-negative tumors [17,18]. As such, HR-positive, HER2-low BC has a distinct molecular profile than HR-negative, HER2-low BC: while the first is enriched with luminal subtypes, the latter demonstrates a predominance of the basal-like subtype, underlining major genetic, clinicopathological, and prognostic differences within the group [17].

Though treated in the same manner as patients with HER2 IHC 0+ BC, patients with HER2-low breast cancer may portray a clinical picture closer to that of HER2-positive BC patients: in a large prospective series, patients with HER2 IHC 2+, FISH-negative BC tended to present with larger tumor size, higher histopathological grade, higher Ki67, and more frequently with axillary nodal involvement [19]. Likewise, disease-free survival of these patients was inferior to that of patients with HER2 0+ and, after the introduction of adjuvant trastuzumab, inferior to that of those with HER2-positive BC. These findings were, to different degrees, replicated in additional series and further defined a subgroup of patients within the HER2-negative space with tumors with HER2 expression levels sufficient enough to exert some oncogenic effect, ultimately putting them in need of additional therapies [20,21].

The targeted treatment options for patients with HER2-low BC will either end upon failure of sequenced endocrine therapies (ET; tamoxifen, aromatase inhibitors or fulvestrant with or without cyclin-dependent kinase 4 and 6 inhibitors or everolimus or alpelisib), in case of HR positivity or are restricted to a subset of those with HR negativity (namely immunotherapy for patients whose tumors are positive for programmed death-ligand 1 (PD-L1) receptor expression or poly(ADP-ribose) polymerase inhibitors for those with germline BRCA mutations) [11,12]. Reliance on traditional cytotoxic chemotherapies thus ensues, a strategy with known constraints, namely dose-limiting, cumulative toxicities and limited survival gains [11,22]. Extending the survival benefits seen with anti-HER2 agents to this expressive parcel of patients is, therefore, an attractive venue, given the poor outcomes of triple-negative BC (TNBC) patients and of endocrine-resistant, HR-positive BC patients [11,23].

In the past, trastuzumab has failed to improve the outcomes of patients with HER2-low BC, and the concept of anti-HER2 agents in this setting was put on hold [18]. Fortunately, this treatment paradigm has recently been re-challenged in light of the promising efficacy results seen with novel and more potent anti-HER2 agents in HER2-low metastatic BC [24,25,26]. It is, therefore, the objective of this article to review the previous body of data and upcoming evidence for the new wave of treatments that may revolutionize the care of HER2-low BC patients. From trastuzumab to trastuzumab–emtansine, moving to trastuzumab–deruxtecan and combinations with immunotherapy, endocrine therapies, cyclin-dependent kinase inhibitors, among others, preclinical, clinical, and safety data supporting further testing of anti-HER2 drugs for the treatment of HER2-low BC patients are reviewed and put into perspective below.

## 2. Rationale for Targeting HER2-Low BC with Anti-HER2 Agents

HER2-low BC cell lines express a considerable quantity of targetable HER2 [27]. In this regard, though it mechanistically made sense that anti-HER2 agents could have clinical applications beyond HER2-positive BC, this was never achieved in the first two decades of experience with HER2-targeted monoclonal antibodies (trastuzumab and pertuzumab), anti-HER2 vaccine (nelipepimut-S), and the first anti-HER2 antibody-drug conjugate (ADC) (trastuzumab–emtansine) [18,28,29,30].

### 2.1. Trastuzumab

Trastuzumab was once hypothesized to work in early-stage BC with minimum levels of HER2 expression: other than blocking the HER2 growth signaling pathway, trastuzumab also causes antibody-dependent cellular cytotoxicity (ADCC). ADDC could play a bigger role in the micrometastatic (adjuvant) setting, where the bulky of disease and tumor-induced immunosuppression are lower than in the metastatic setting; thereby, the level of HER2 expression in the tumor is less important for trastuzumab activity [19,20]. Moreover, in the micrometastatic setting, mice xenograft models of HER2-negative luminal BC have been shown to have their implants’ growth driven by HER2 upregulation in the bone, a common site of metastatic seeding. When the mice were given trastuzumab shortly after tumor implantation, despite the original absence of HER2 overexpression in the model, tumor growth could be halted, forming the basis to postulate an adjuvant effect of trastuzumab on HER2-negative disease [31].

In the clinical setting, post hoc retrospective analysis of two phase 3 trials further corroborated this hypothesis. In NSABP B-31 and NCCTG N9831, comparing adjuvant chemotherapy with trastuzumab vs. chemotherapy alone, patients were initially eligible based on local laboratory HER2 assessment. Discordant cases, where final tumor results for HER2 were actually negative upon central pathology review, were thus included [32]. In B-31, 9.7% of patients were centrally-assessed as HER2-negative, and yet they derived a beneficial effect from trastuzumab (relative risk for disease-free survival (DFS), 0.34; 95% CI, 0.14 to 0.80), similarly to HER2-positive patients [33]. Moreover, HER2 messenger RNA levels were consistently lower in the HER2-negative tumors compared to the HER2-positive tumors, providing further evidence that HER2-negativity at the central pathology review was not a false-negative result at the transcriptomic level and yet patients were benefiting from trastuzumab. In N9831, among 5.5% centrally assessed HER2-negative BC, a trend towards benefit with trastuzumab was found (hazard ratio (HR) for DFS, 0.51; 95% CI, 0.21–1.23) [34].

Despite these early positive signals, NSABP B-47, a large randomized phase 3 trial that enrolled 3270 patients with HER2-low BC to adjuvant trastuzumab plus chemotherapy vs. chemotherapy alone, failed to prove any beneficial effect of trastuzumab (HR for invasive DFS, 0.98; 95% CI, 0.76 to 1.25) (Table 1) [18]. In subgroup analyses, even patients with a higher degree of HER2 expression (IHC 2+) did not benefit from trastuzumab, similarly to patients with HER2 IHC 1+ tumors.

### 2.2. Pertuzumab

Pertuzumab is another monoclonal antibody targeted against HER2, which prevents its homodimerization and also heterodimerization with other activating HER family partners, further blocking downstream growth signaling activation [39]. Unlike trastuzumab, pertuzumab is capable of inhibiting tumor growth of xenograft models even in the absence of HER2 overexpression [40]. Nonetheless, pertuzumab alone, given for previously treated HER2-negative or low BC patients, yielded disappointing tumor responses in a phase II trial (Table 1) [28].

### 2.3. Nelipepimut-S

E75 is a HER2-derived peptide capable of stimulating CD8+ T cytotoxic lymphocytes to recognize and eliminate HER2-expressing cancer cells [41]. Combined with an immunoadjuvant (granulocyte macrophage-colony stimulating factor (GM-CSF)), the HER2-targeting vaccine nelipepimut-S has been shown to induce E75-specific CD8+ T-cells expansion, which is even greater in patients with HER2-low BC [42]. Given with trastuzumab, the expansion of E75-specific CD8+ T-cells is amplified, underlying a synergism for the combination [43].

In this sense, an early immunotherapeutic approach tested in clinical trials of HER2-low BC patients was nelipepimut-S combined with trastuzumab. It was evaluated after standard adjuvant therapy for patients with HR-negative/HER2 IHC 1+ or 2+ (FISH-negative), node-negative BC or node-positive BC regardless of HR status [29]. Patients were randomly assigned to receive trastuzumab once every 3 weeks for 1 year and placebo with GM-CSF (control arm; *n* = 139) or nelipepimut-S (experimental arm; *n* = 136). In the intention-to-treat analysis, no statistical difference was observed for the primary endpoint (24-month DSF-rate of 89.9% in the vaccine arm vs. 83.8% in the control arm (HR = 0.62; 95% CI = 0.31–1.25)), albeit in the subgroup of patients with HR-negative BC, nelipepimut-S was able to significantly improve it (Table 1). Still, nelipepimut-S development for HER2-low BC did not move forward.

### 2.4. Trastuzumab-Emtansine

Trastuzumab–emtansine (T-DM1) is a 2nd generation ADC composed of the HER2-targeting vehicle trastuzumab, bound via a non-cleavable thioether linker to the potent anti-tubulin, maytansine derivative DM1, with a drug–antibody ratio of 3.5:1. Its antitumor properties reside not only on the blockade of the HER2 signaling pathway and ADCC induction by trastuzumab but also on the internalization of the cytotoxic moiety by HER2 expressing cells, therefore, having a more potent cytotoxic effect within tumor cells instead of on healthy tissues (i.e., a better therapeutic index than traditional cytotoxic drugs) [44].

Unlike trastuzumab, T-DM1 was never prospectively tested in HER2-low BC. Nonetheless, in two phases 2 trials testing the efficacy and safety of T-DM1 in HER2-positive metastatic BC patients previously treated with at least trastuzumab, retrospective, exploratory analyses according to central laboratory assessment of HER2 status found poor clinical activity of T-DM1 among patients with HER2-normal BC compared to patients with HER2-positive BC (Table 1) [35,36].

T-DM1 was, however, prospectively tested in the analogous setting of HER2-positive but heterogeneous BC [30]. Genetic heterogeneity is present in a significant proportion of BCs that would otherwise be classified as HER2 FISH-negative [45]. In a phase 2 study of neoadjuvant T-DM1 plus pertuzumab, HER2 heterogeneity was found in 10% of cases (16 out of 157 enrolled patients). No patients with HER2 heterogeneity achieved a pathological complete response (pCR), whereas 55% of those with homogeneous tumors did it. Altogether, T-DM1 is hypothesized to fare poorly if tested in the HER2-low setting.

Given this apparent failure of the early anti-HER2 agents for the treatment of HER2-low BC, why should this niche be revisited with other anti-HER2 agents? Which are the anti-HER2 agents capable of targeting HER2-low BC cells with clinical efficacy? Are there combinations capable of overcoming those low levels of HER2 expression?

## 3. Mechanisms of Action and Clinical Efficacy of the Novel Anti-HER2 Drugs in HER2-Low BC

The novel agents being tested for the treatment of HER2-low BC patients are categorized as ADCs deploying anti-HER2 epitopes in their monoclonal antibody component, though with different cytotoxic warheads than trastuzumab–emtansine, and a bi-specific antibody targeting HER2 and HER3 [46,47,48,49]. These novel agents have differential features that may explain their in vitro and in vivo activity beyond that seem with earlier anti-HER2 agents, such as the bystander killing effect of non-target cancer cells with the novel ADCs, and the bypass of HER3-mediated resistance with zenocutuzumab, the bi-specific antibody [49,50]. Therefore, their deployment in clinical trials of HER2-low BC is further explored.

### 3.1. Trastuzumab-Deruxtecan

**Figure 1:**
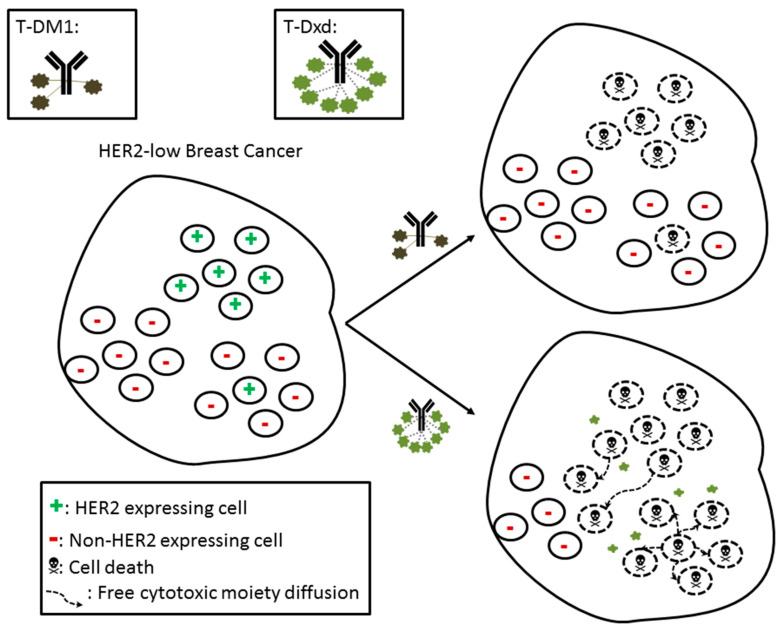
Schematic representation of HER2-low breast cancer being exposed to trastuzumab–emtansine (T-DM1; top of figure), with its non-cleavable linker attaching trastuzumab to the cytotoxic maytansine derivative, and to trastuzumab–deruxtecan (T-Dxd; the bottom of figure), with its cleavable linker attaching trastuzumab to the diffusible, cytotoxic exatecan derivative. While DM1 is trapped inside the trastuzumab-targeted cells, Dxd is freely diffusible and able to kill non-expressing HER2-cells. Ultimately, the bystander killing effect represented here explains the success of T-Dxd (and also of trastuzumab–duocarmazine) in targeting HER2-low tumours, despite their lower degree and heterogeneity of HER2 expression.

Trastuzumab–deruxtecan (T-Dxd) is an ADC, which, apart from sharing the same HER2 targeting monoclonal antibody, differs from T-DM1 in several aspects (Table 2). Most importantly, its cleavable linker, with its cell membrane permeable exatecan derivative (a topoisomerase I inhibitor payload) altogether with a higher drug-antibody ratio (of 8:1), elicits a greater antitumor effect [46]. There are two fronts for that: first, a greater amount of the cytotoxic payload reaches the targeted cells (i.e., more potent cytotoxic effect); second, there is the bystander killing effect. This effect can be understood as the potential (of a given ADC) to kill the antigen-negative, surrounding cells of a targeted antigen-positive cell, following diffusion of the free cytotoxic moiety from inside the dead, antigen-positive cells [50]. With that, low/non-expressing HER2 cells in the tumor vicinity of HER2 overexpressing cells are also affected by T-Dxd, cell-membrane permeable cytotoxic moiety (Figure 1) [51]. Ultimately, the bystander killing effect has the potential to induce an improved clinical activity in the setting of low or heterogeneous HER2 expression while maintaining a safe therapeutic index.

In fact, T-Dxd is not only active in a T-DM1 insensitive, HER2-positive patient-derived xenograft model, but it also retains its activity against several BC models with low levels of HER2 expression, unlike T-DM1 [46]. Both preclinical observations have been paralleled in early phase trials, with meaningful clinical responses seen in a T-DM1-refractory, HER2-positive BC patients cohort and also in a heavily pretreated cohort of HER2-low BC patients [24,52]. In the latter trial, on its dose expansion part, 54 HER2-low metastatic BC patients were enrolled. The ORR was 37% (95% CI, 24.3–51.3%), with a median duration of response of 10.4 months (Table 1). T-DXd activity was seen across multiple subgroups, including HER2 IHC status (2+ and 1+), HR status, previous HER2-targeting therapy, and prior exposure to a CDK4/6i. Based on these promising efficacy signals, two randomized, phase III trials comparing T-DXd versus chemotherapy of physician’s choice in HER2-low, unresectable or metastatic BC are currently ongoing [53,54].

### 3.2. Trastuzumab-Duocarmazine

Trastuzumab–duocarmazine (SYD985) is a novel ADC in earlier phases of clinical development, composed of trastuzumab, a cleavable linker, and a DNA-alkylating duocarmycin warhead [47]. Its drug–antibody ratio is 2.8:1, and the cytotoxic moiety is actually a cell-permeable pro-drug (*seco*-duocarmycin-hydroxybenzamide-azaindole (*seco*-DUBA)), cleaved into the active toxin (DUBA) in intracellular lysosomes by proteases, following internalization. Interstitial cleavage of trastuzumab–duocarmazine by malignant cells secreting cathepsin B also occurs, generating free DUBA capable of inducing the desired bystander killing effect (Table 2) [50,55]. Despite its lower drug–antibody ratio, trastuzumab–duocarmazine has been shown significantly more potent than T-DM1 in comparative essays of HER2-low, patient-derived xenograft BC models [47].

Early clinical signs of efficacy with trastuzumab–duocarmazine have already been demonstrated in all levels of HER2 IHC expression in a first-in-human trial, albeit thus far the only phase 3 trial recruiting is restricted to HER2-positive MBC patients (clinicaltrial.gov identifier: NCT03262935) [25]. On its first-in-human study, trastuzumab–duocarmazine was tested on treatment-refractory, locally advanced or metastatic tumors in order to assess safety, pharmacokinetics and preliminary tumor activity. Forty-seven HER2-low BC patients were enrolled in the BC dose expansion cohort. Six of 15 HR-negative (40%, 95% CI 16.3–67.6) and 9 of 32 (28%, 95% CI 13.8–46.8) HR-positive patients achieved a partial response (Table 1).

### 3.3. XMT-1522

XMT-1522 inaugurated a generation of HER2-targeted ADCs with distinct structural features: instead of trastuzumab, XMT-1522 uses HT-19, which binds a different HER2 epitope than trastuzumab, linked via a biodegradable cysteine-linkage to an auristatin-derivative, yet another anti-tubulin agent. XMT-1522 has the highest drug–antibody ratio of the field (12:1), elicits a controlled bystander killing effect, and has been shown to outperform T-DM1 in HER2-positive and low xenograft cancer models [48]. Despite preliminary signs of clinical efficacy, XMT-1522 development was halted at phase I due to commercial reasons [56].

### 3.4. Zenocutuzumab

Not only ADCs are being tested in HER2-low metastatic BC patients. Currently, the bi-specific humanized IgG1 antibody zenocutuzumab (MCLA128) is undergoing clinical development, with results from phase II trials available [26,57]. With the novel dock and block effect, zenocutuzumab works by docking to HER2 domain I, positioning the anti-HER3 arm fit to block the domain III of HER3, such as to prevent the binding of any activating ligand (e.g., heuregulin) [49]. HER2/HER3 heterodimerization is, therefore, inhibited altogether with the subsequent oncogenic intracellular signaling cascade. Zenocutuzumab is more potent than pertuzumab in inhibiting HER2/HER3 heterodimerization, including at higher heuregulin concentrations, and is also capable of eliciting ADCC [49].

In HR-positive/HER2-low BC, bidirectional crosstalk between the estrogen receptor (ER) and the HER2/HER3 axis can drive ET resistance, whereby upregulation of heuregulin and HER2/HER3 heterodimers can phosphorylate the ER, and ER signaling can upregulate HER2 and HER3 expression [18,39]. In this sense, zenocutuzumab with ET has been demonstrated to sustain a better antitumor effect than ET alone in HER2-low BC xenograft models [49]. In a phase I/II trial enrolling HER2-positive BC and other epithelial tumors, the zenocutuzumab recommended phase II dose was determined and shown to be remarkably safe, with very few grade 3–4 adverse events (AEs). Afterwards, this dose was tested in a phase 2, single-arm trial of endocrine-resistant, ER-positive/HER2-low BC patients, who had experienced disease progression while on a cyclin-dependent kinase 4 and 6 inhibitor (CDK4/6i) [26,57]. Fifty patients were treated with zenocutuzumab plus ET (fulvestrant or an aromatase inhibitor), and 8 sustained a clinical benefit at week 24 (clinical benefit rate of 16.7% (90% CI, 8.6–28.1)), with one patient showing a partial response followed by a long-lasting disease stabilization (Table 1).

## 4. Exploiting Combination Treatments in HER2-Low Breast Cancer

Combination strategies in HER2-low BC with anti-HER2 agents that are currently being tested include immune checkpoint inhibitors (ICIs), CDK4/6i, ET, among others. This effort includes both the deployment of novel anti-HER2 agents as well as the repurposing of older drugs.

### 4.1. Immunotherapy

Preclinical and clinical data suggest that HER2-positive tumors are immunogenic [58]. HER2-positive tumors have a higher mutational burden (2.05 mutations/Mb) compared to luminal tumors (up to 1.38 mutations/Mb) [59]. They also contain a higher number of tumor-infiltrating lymphocytes (TILs) and higher PD-L1 expression [58,59]. The lymphocytic infiltrate observed in primary HER2-positive tumors has been associated with pCR and improved survival outcomes [60]. Moreover, ADCC is a key player when it comes to modulating the effect of trastuzumab [58]. Preclinical studies suggest that the combination of trastuzumab with drugs targeting immune checkpoints could overcome trastuzumab resistance [60]. High expression of programmed cell death protein 1 (PD-1), PD-L1 and other checkpoint molecules has been observed in this setting [58].

Several chemotherapeutic agents, by inducing tumor cell death and immunogenic cell death, also activate the immune system [61]. Topoisomerase inhibitors have successfully been combined with ICIs in syngeneic mouse models, but there is a concern that they could induce lymphopenia and thus attenuate the effect of ICIs [61]. In this sense, T-Dxd could be an ideal partner for ICI by combining trastuzumab-induced ADCC with the topoisomerase I inhibitor warhead-induced immunogenic cell-death and the improved therapeutic index [62]. In preclinical studies, T-Dxd increased tumor-infiltrating dendritic cells, upregulated their CD86 expression in vivo, increased tumor-infiltrating CD8+ T cells and enhanced PD-L1 and major histocompatibility complex class I expression on tumor cells [62]. Furthermore, in mouse models, combination therapy with T-Dxd and an anti-PD-1 antibody was more effective than monotherapy, possibly due to increased T-cell activity and upregulated PD-L1 expression induced by T-Dxd [62].

In a phase 1b study, 16 patients with treatment-refractory, HER2-low BC were treated with the combination of T-Dxd and the anti-PD-1 nivolumab [38]. Overall, treatment with T-Dxd + nivolumab had a manageable safety profile and showed preliminary encouraging efficacy data, albeit similar to that of T-Dxd monotherapy (Table 1).

Altogether, despite preliminary, these data support the rationale for combining anti-HER2 therapies with ICI in trials of HER2-low BC patients (Table 3).

### 4.2. Endocrine Therapies

Building upon the hypothesis of tumor resistance development via bidirectional crosstalk between HER2/HER3 and ER axes, Collins et al. used a xenograft mouse model of ER-positive/HER2-low human BC to evaluate a triple therapy targeting HER2, HER3, and ER [63]. It was found that the addition of fulvestrant significantly enhanced antitumor responses to the HER2 and 3 targeting agents. In this sense, as mentioned previously, the addition of the HER2/HER3 bi-specific antibody zenocutuzumab to ET showed clinical activity in ET and CDK4/6i-refractory patients, resulting in an endocrine-sensitivity rescue of 17% of them (Table 1) [26].

Co-targeting of the ER and HER2 axis is also being attempted in a phase Ib trial with T-Dxd and anastrozole or fulvestrant [64].

### 4.3. CDK4/6i

As a downstream pathway to HER2, deregulation of the cyclin D1-CDK4 axis is a common mediator of HER2 therapy resistance [65]. Moreover, preclinical evidence suggests that CDK4/6i may hold activity beyond luminal BC: not only CDK4/6i are effective in lapatinib-resistant, HER2-positive cell models, but their use altogether with anti-HER2 agents has a synergistic effect [66,67]. CDK4/6i sensitizes lapatinib-resistant cell lines to HER2-targeted therapies, leading to better inhibition of cell proliferation and, in patient-derived xenograft tumors and transgenic mice models, to improved control of tumor growth and delays tumor recurrences, respectively.

In order to test a chemotherapy-free approach in the early disease setting, the NA-PHER2 trial, an open-label, exploratory, phase II study, evaluated neoadjuvant pertuzumab, trastuzumab, fulvestrant and the CDK4/6i palbociclib [37]. It included 23 patients with HR-positive/HER2-low BC with a Ki67 > 20% in one of its cohorts (cohort C), with 2 co-primary endpoints: on-treatment changes of baseline Ki67 to week 2 and at surgery (16 weeks). A robust Ki67 decrease was demonstrated, especially at week 2, as well as 18 (78.3%) objective responses were seen, underlining the clinical potential of the combination. However, unlike cohort A and B, where only HER2-positive BC cases were enrolled, no patient achieved a pCR (Table 1) [68].

### 4.4. Other Combinations

A few other treatment combinations are being tested with T-Dxd in phase 1, multicohort clinical trial, including chemotherapy agents and AKT inhibitors, based on the premise of distinct and non-overlapping, cytotoxic mechanisms resulting in additional efficacy [64]. Hitherto, phase 1 data are still being generated in order to evaluate the safety of these distinct combinations (clinicaltrial.gov identifier: NCT04556773).

## 5. Safety

**Figure 2:**
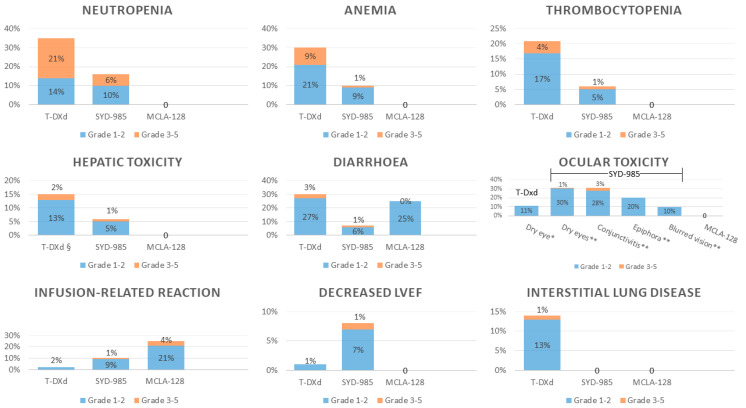
Incidence of key adverse events induced by trastuzumab–deruxtecan, trastuzumab–duocarmazine and zenocutuzumab. Abbreviations: T-DXd: trastuzumab–deruxtecan, SYD-985: trastuzumab–duocarmazine, MCLA-128: zenocutuzumab, LVEF: left ventricular ejection fraction. § Data extracted from all enrolled patients (dose-escalation and dose-expansion parts). * The only reported ocular adverse event was dry eye (data extracted from all enrolled patients (dose-escalation and dose-expansion parts)). ** other non-reported ocular adverse events include episcleritis (3%), corneal toxicity (1%) and retinal hemorrhage (1%).

The safety profile of the new different compounds tested for HER2-low BC is interestingly heterogeneous, according to the cytotoxic warhead, in the case of ADCs, or to the immunogenicity capacity, in the case of bispecific antibodies (Figure 2). Thus, upon regulatory approval of these novel agents, clinicians will be able to choose according to patient comorbidities and preferences in case of comparable efficacy.

### 5.1. Hematological Toxicity

Despite their targeted cytotoxicity, all ADCs share some hematological AEs [69]. In the phase 2 study on T-Dxd, the following all-grade hematological AEs were reported: neutropenia (35%), anemia (30%) and thrombocytopenia (21%) [52]. Grade 3 or higher hematological AEs were observed for neutropenia (20%), anemia (8%), leukopenia (6%) and thrombocytopenia (4%). Febrile neutropenia occurred in 2% of patients. In the dose-expansion part of the phase 1 study of trastuzumab–duocarmazine, there were neutropenia (10% grade 1–2 and 6% grade 3), anemia (9% grade 1–2 and 1% grade 3), thrombocytopenia (5% grade 1–2 and 1% grade 3), lymphopenia (5% grade 1–2 and 1% grade 3) and pancytopenia AEs (1% grade 3) [25].

### 5.2. Hepatic Toxicity

All ADCs, including those already used in clinical practice, such as T-DM1, are potentially hepatotoxic [70,71]. In the phase 2 study of T-DXd, AST and ALT increase was observed in 15% and 12% of patients (all-grade), respectively; grade 3 elevations occurred in 2% of cases [52]. In the phase I dose-expansion part of trastuzumab–duocarmazine, hepatic AEs were AST increase (5% grade 1–2 and 1% grade 3), GGT increase (3% grade 1–2, 1% grade 4) and alkaline phosphatase increase (2% grade 1–2 and 1% grade 3) [25]. One patient died from hepatic failure, although this death was not considered treatment-related.

### 5.3. Gastrointestinal Toxicity

Due to its topoisomerase inhibitor warhead, T-Dxd is characterized by a comparatively worse gastrointestinal (GI) toxicity profile [72]. Gastrointestinal AEs were reported among the most common T-Dxd-induced AEs and included nausea (78% all grades, 8% grade 3), vomiting (46% all grades, 4% grade 3), constipation (36% all grades), decreased appetite (31% all grades, 2% grade 3), diarrhea (29% all grades, 3% grade 3) and abdominal pain (17% all grades, 1% grade 3) [52]. Regarding trastuzumab–duocarmazine, the most common GI AEs were decreased appetite and nausea, reported in less than 20% of patients; the only grade 3 gastrointestinal AEs reported were diarrhea and decreased appetite (both 1% in the dose-expansion cohort) [25].

### 5.4. Pulmonary Toxicity

Albeit uncommon, interstitial lung disease (ILD) is potentially life-threatening, and patients treated with HER2-targeting ADCs should be carefully monitored, according to the previous experience with T-DM1 [70,71]. Likewise, in a phase 1b trial, three toxic deaths occurred with T-DXd: one case of ILD and two cases of pneumonitis. Globally, eleven ILD were centrally re-evaluated, and eight of them were considered T-DXd-induced. The most common AEs leading to treatment discontinuation were pneumonitis (*n* = 7) and ILD (*n* = 3). Grade 2 or higher ILD were treated with steroids [24]. In the phase 2 study of T-DXd in HER2-positive BC, ILD was reported for 14% of patients [52]. In a pooled analysis of ILD data coming from seven trials of T-DXd, among 665 patients, 66 (9.9%) developed ILD, with 13 (2%) of grade ≥3, 5 (<1%) deaths, and with a median (range) time to onset of 149 days (16–596 days) [73]. Most grade ≥2 cases were managed with steroids or steroids plus antibiotics. A higher dose of T-Dxd (6.4 mg/kg, instead of the recommended phase II dose of 5.4 mg/kg) and Japanese origin seem to be risk factors for ILD.

In the dose-escalation phase for trastuzumab–duocarmazine, one fatal pneumonitis was reported as a dose-limiting toxicity (2.4 mg/kg), but the risk of pneumonitis diminished at the recommended phase 2 dose of 1.2 mg/kg q3w [25]. Further analyses are required to better understand the underlying mechanism and risk factors for the novel ADC-induced ILD, as well as how to properly manage it.

### 5.5. Ocular Toxicity

Ocular toxicities dominate the toxicity profile of trastuzumab–duocarmazine [25]. Conjunctivitis was reported in up to 30% of patients in the dose-expansion part of the phase 1 trial. Other reported ocular AEs were dry eyes, keratitis and blurred vision. Four patients (3%) had grade 3 conjunctivitis in the dose-expansion cohort. Dose-reduction, decrease in the frequency of administration, or prophylactic use of eye drops did not seem to impact these AEs. Nonetheless, most patients were able to continue trastuzumab–duocarmazine and most ocular problems were reported to improve, therefore, suggesting that these AEs are manageable.

### 5.6. Cardiotoxicity

Cardiotoxicity represents a potential risk for patients treated with new HER2-targeting agents, based on the previous experience gathered from older anti-HER2 agents [74]. Briefly, HER2-rich cardiomyocytes rely on HER2 growth signaling to maintain their homeostasis and endure oxidative stress, such as that induced by anthracyclines, for which most BC patients are exposed through the course of their disease. Upon blockade of this important survival pathway, cardiotoxicity may follow, which includes mainly decreases in left ventricular ejection fraction (LVEF). QT interval prolongation has also been reported. In the phase II study of T-DXd on HER2-positive metastatic BC, LVEF decrease incidence was low (1.6%): two patients had grade 2, and one had a grade 3 event. All patients recovered after treatment interruption. QT interval prolongation of any grade was reported for 9 patients (4.9%) and of grade 3 in 2 patients (1.1%) [52]. In the dose-expansion phase I study of trastuzumab-duocarmazine, an LVEF decrease was observed in 10 patients (7%) with grade 1–2 and in 1 patient (3%) with grade 3. In 8 patients (5%), an absolute decrease of at least 10% from baseline to a value below 50% was reported [25].

With zenocutuzumab, no treatment-related cardiotoxicities of clinical relevance were reported in both early phase trials [26,57]. Although the number of treated patients is smaller comparing to the trastuzumab-containing ADCs, this encouraging cardiac safety data likely reflects the improved in vitro cardiomyocyte viability with zenocutuzumab, as compared to trastuzumab [49].

### 5.7. Neuropathy

Unlike with the anti-tubulin containing T-DM1, neuropathy seems rare with the novel ADCs [70,71]. This is due to the different mechanism of action of their cytotoxic moieties, which does not induce functional disruption of the microtubules in the peripheral neurons.

### 5.8. Infusion-Related Reactions

Infusion-related reactions (IRR) spam mild reactions, like pyrexia, rash, and flushing, to severe reactions, i.e., overt anaphylactic shock [75]. They seem to be more frequent with zenocutuzumab than with the novel ADCs, for which the overall incidence of IRR is less than 10% [26,57].

In the early phase trials of zenocutuzumab, all-grade IRR incidence ranged from 18% to 36%, with only a few grade 3–4 IRR (4%). Nonetheless, this has prompted the mandatory use of prophylactic anti-histaminic, anti-pyretic, and corticosteroid prior to zenocutuzumab infusion once the recommended phase 2 dose was determined.

## 6. Conclusions

Three decades after the characterization of the oncogenic HER2 protein, anti-HER2 therapies have just started to advance towards the field of HER2-low BC treatment. International guidelines currently recommend a binary model (HER2-positive vs. negative) to guide clinicians in treatment decisions. However, a great proportion of patients (≈40–50%) classified as HER2-negative are, in fact, HER2-low, a population at a high unmet medical need. Despite past drawbacks with older drugs, a new generation of anti-HER2 agents has recently shown encouraging signs of clinical activity and safety in HER2-low disease.

Due to the retention of all trastuzumab antitumor properties, associated with an improved tumor-specific cytotoxic effect, as well as the bystander killing effect, ADCs like T-Dxd and trastuzumab–duocarmazine are able to target and kill HER2-expressing BC cells even upon low-level of HER2 expression, a once limiting step for the clinical activity of anti-HER2 agents.

Despite some undisputed successes and promising expectations coming from the new anti-HER2 agents in HER2-low BC, to the point where two phase 3 trials are already ongoing, this new treatment strategy underlines the steep road of cancer drug development, characterized by complex technologies, the important commitment of multiple stakeholders and, oftentimes, clinical outcomes not always fulfilling preclinical expectations.

Albeit having an improved therapeutic index than traditional chemotherapies, anti-HER2 ADCs still retain some class-related AEs, many in common with the general profile of chemotherapeutic agents (myelotoxicity, hepatotoxicity) and trastuzumab profile (cardiotoxicity), but some depending on the class of the cytotoxic warhead (mainly GI toxicity with T-Dxd and ocular toxicity with trastuzumab–duocarmazine), altogether with potentially life-threatening AEs, such as ILD. A better understanding of the pathophysiology of such AEs, altogether with the delineation of risk factors, prevention, and treatment measures, will further improve the safety profile of these ADCs.

Overall, T-Dxd may be the first HER2-targeted therapy approved for HER2-low BC patients, based on the strong preclinical rationale, encouraging early efficacy signs hitherto discussed, and its current stage of development. To overcome the current standard of care, other than leveraging what has been observed in early phase trials, the two-phase 3 clinical trials currently ongoing must be able to decrease the occurrence and severity of ILD thus far observed with T-Dxd, with replicability in the real world.

A further step in the development of HER2-low BC treatment is coming from the evaluation of new treatment combination strategies. Considering ADC’s ability to induce immunogenic cell death and thereby an immune-hot tumor microenvironment, further results from studies evaluating ADCs in combination with ICIs are eagerly expected. Combined with ET, new anti-HER2 agents, like zenocutuzumab, could provide a new, chemotherapy-free approach for patients with endocrine-resistant HER2-low BC.

In summary, where trastuzumab and older anti-HER2 agents have left a niche, the new anti-HER2 agents may succeed. Collectively, these early trial results are building the foundations for the exciting new field of HER2-low BC treatment.

## Figures and Tables

**Table 1:** Key clinical efficacy data of anti-human epidermal growth factor receptor-2 (HER2) agents in HER2-low BC.

Author	Study Design	Study Population	N	Treatment	Main Efficacy Results
**Single anti-HER2 agents**					
Fehrenbacher et al. NSABP B-47 [18]	Phase 3, randomized (1:1) trial	High-risk early BC-negative for HER2 by FISH and with IHC 1+ or 2+	3270	Adjuvant ChT with or without trastuzumab	5-year iDFS: 89.8% vs. 89.2%, HR 0.98; 95% CI, 0.76–1.25; *p* = 0.85/ OS: 94.8% vs. 96.3%, HR 1.33; 95% CI, 0.90–1.95; *p* = 0.15
Gianni L et al. [28]	Phase 2, randomized (1:1) trial	HER2-low metastatic BC	78	Pertuzumab (420 mg q3w vs. 1050 mg q3w)	CBR (CR + PR + SD at 24 weeks): 9.8% in 420 mg q3w arm vs. 5.4% in 1050 mg q3w arm Median time to progression: 6.1 weeks (both arms)
Burris et al. [35]	Phase 2, single-arm trial	HER2-positive metastatic BC (including HER2-low BC after central assessment)	112 pts (21 HER2-low)	Trastuzumab emtansine (T-DM1)	ORR: 4.8% (95% CI, 1.0–21.8%) vs. 33.8% (95% CI, 23.2–44.9%) Median PFS: 2.6 mo (95% CI, 1.4–3.9 mo) vs. 8.2 mo (95% CI, 4.4 mo to NE)
Krop et al. [36]	Phase 2, single-arm study	HER2-positive metastatic BC (including HER2-low BC after retrospective re-evaluation)	110 pts (15 HER2-low)	Trastuzumab emtansine (T-DM1)	ORR: 20% (95% CI, 5.7–44.9) vs. 41.3% (95% CI 30.4–52.8) Median PFS: 2.8 mo (95% CI 1.3-NE) vs. 7.3 (95% CI, 4.6–12.3)
Modi et al. [24]	Phase 1, dose-expansion study	HER2-low BC refractory to standard therapies	54	Trastuzumab–deruxtecan (T-DXd) (DS8201a)	ORR: 37% (95% CI, 24.3–51.3%) Median DoR: 10.4 mo (95% CI, 8.8 mo-NE)
Banerji et al. [25]	Phase 1 dose-expansion study	Advanced BC, gastric, urothelial, or endometrial cancer with at least HER2 IHC 1+	146 (47 HER2-low BC)	Trastuzumab duocarmazine (SYD985)	ORR: 28% (95% CI, 13.8–46.8%) in HR+ HER2-low BC, 40% (95% CI, 16.3–67.6%) in HR- HER2-low BC
**Combination therapies**					
Hickerson et al. [29]	Phase II, randomized (1:1), blinded, placebo-controlled	Node-positive (or negative if HR-negative) HER2-low BC patients after standard adjuvant therapy	275	Nelipepimut-S + trastuzumab vs. placebo + GM-CSF + trastuzumab	24-month DFS rate: 89.9% in the vaccine arm vs. 83.8% in the control arm (HR = 0.62; 95% CI = 0.31–1.25; *p* = 0.18); 24-month DFS rate in the subgroup of TNBC: 92.6% vs. 70.2%, respectively (HR = 0.26; 95% CI = 0.08–0.81; *p* = 0.013)
Gianni et al. [37]	Phase II, multicenter, multicohort trial	Cohort C: HR-positive/HER2-low early BC	23	Trastuzumab + pertuzumab + fulvestrant + palbociclib	Baseline mean Ki67: 32.4% Mean Ki67 at week 2: 2.6% (mean a change of −29.5; *p* < 0.001) Mean Ki67 at surgery: 7.5% (mean change of −19.3; *p* < 0.001)
Pistilli et al. [26]	Phase 2 study	ER+/HER2-low metastatic BC refractory to ET/CDK4/6i	50	Zenocutuzumab (MCLA-128) + ET	CBR (CR + PR + SD at 24 weeks): 16.7% (90% CI 8.6–28.1)
Hamilton et al. [38]	2-part, phase 1b study	Cohort 2: HER2-low BC after standard therapy	16	Trastuzumab–deruxtecan + nivolumab	Confirmed ORR by independent central review: 38% (95% CI, 15–65); DoR not evaluable

Legends: BC: breast cancer; CBR: clinical benefit rate; CDK 4/6i: cyclin-dependent kinase 4/6 inhibitors; CI: confidence interval; CR: complete response; DoR: duration of response; ER: estrogen receptor; ET: endocrine therapy; GM-CSF: granulocyte macrophage-colony stimulating factor; HR: hazard ratio; iDFS: invasive disease-free survival; IHC: immunohistochemistry; NE: not evaluable; ORR: overall response rate; NSCLC: non-small cell lung cancer; PFS: progression-free survival; PR: partial response; RFI: relapse-free interval; SD: stable disease.

**Table 2:** Comparison of trastuzumab–emtansine (T-DM1) vs. trastuzumab–duocarmazine (SYD−986) vs. trastuzumab–deruxtecan (T-Dxd).

Antibody-Drug Conjugate	T-DM1	SYD-986	T-Dxd
HER2 targeting vehicle	Trastuzumab	Trastuzumab	Trastuzumab
Linker	Non-cleavable	Cleavable	Cleavable
Drug–antibody ratio	3.5:1	2.8:1	8:1
Cytotoxic moiety	Maytansine derivative	Seco-DUBA	Exatecan derivative
Cytotoxic moiety MoA	Antimicrotubule (mitotic poison)	Alkylating agent	Topoisomerase I inhibitor
Diffusible cytotoxic moiety?	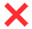	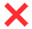	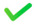
Bystander killing effect?	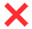	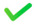	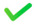
Targets HER2-positive or homogenous tumors?	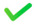	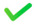	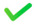
Targets HER2-low or heterogeneous tumors?	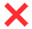	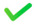	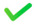

Legend: MoA = mechanism of action.

**Table 3:** Ongoing combinations trials with immune checkpoint inhibitors and the novel anti-HER2 agents in HER2-low breast cancer.

Drugs Tested	Study Design	Patient Population	Primary Endpoint	Status	ClinicalTrials.gov Identifier
Trastuzumab–deruxtecan + pembrolizumab	Phase Ib, open-label, two-part, multicenter, nonrandomized, multiple-dose	Advanced BC (HER2-positive and HER2-low) and HER2-positive NSCLC	Phase I: MTD Phase II: ORR	Recruiting	NCT04042701
Trastuzumab–deruxtecan + nivolumab	Phase Ib, multicenter, two-part, open-label	Advanced BC (HER2-positive and HER2-low) and urothelial cancer	Phase I: MTD Phase II: ORR	Ongoing	NCT03523572
Trastuzumab–deruxtecan + durvalumab	Phase Ib/II, two-stage, open-label, multicenter	Arm 6: Advanced TNBC with low HER2 expression	Safety	Recruiting	NCT03742102
Trastuzumab–deruxtecan + durvalumab + paclitaxel	Phase Ib, open-label, modular, dose-finding and dose-expansion	Module 2: advanced HER2-low BC	Safety and tolerability	Not yet recruiting	NCT04556773

Legend: BC: breast cancer; ET: endocrine therapy; NSCLC: non-small cell lung cancer; MTD: maximum tolerated dose; ORR: objective response rate; TNBC: triple-negative breast cancer.

## Data Availability

No new data were created or analyzed in this study. Data sharing is not applicable to this article.

## Abbreviations

ADC: antibody-drug conjugate; BC: breast cancer; CBR: clinical benefit rate; CDK 4/6i: cyclin-dependent kinase 4/6 inhibitors; CI: confidence interval; CR: complete response; DoR: duration of response; ER: estrogen receptor; ET: endocrine therapy; GM-CSF: granulocyte macrophage-colony stimulating factor; HR: hazard ratio; iDFS: invasive disease-free survival; IHC: immunohistochemistry; LVEF: left ventricular ejection fraction; MCLA-128: zenocutuzumab; MoA: mechanism of action; MTD: maximum tolerated dose; NE: non-evaluable; NSCLC: non-small cell lung cancer; ORR: overall response rate; PFS: progression-free survival; PR: partial response; RFI: relapse-free interval; SD: stable disease; SYD−986: trastuzumab–duocarmazine; T-DM1: trastuzumab–emtansine; T-Dxd: trastuzumab–deruxtecan; TNBC: triple-negative breast cancer.
